# A retrospective investigation of the pmx-dhp's efficacy in 88 severe sepsis cases

**DOI:** 10.1186/2197-425X-3-S1-A794

**Published:** 2015-10-01

**Authors:** M Tanaka, K Ueda, Y Kawazoe, T Yonemitsu, M Kida, Y Shima, S Yamazoe, Y Iwasaki, S Kato, Emergency and Critical Care Medicine

**Affiliations:** Wakayama Medical College, Wakayama City, Japan

## Objective

To investigate the efficacy of polymyxin-B direct hemoperfusion (PMX-DHP) in terms of improving clinical conditions and life prognosis in severe sepsis cases.

## SUBJECTS AND METHODS

The subjects were 88 cases admitted to our hospital ICU between April 2010 and July 2014 with the diagnosis of severe septic shock requiring continuous norepinephrine injection (Cathecholamine Index≧10). We investigated PMX-DHP efficacy by retrospectively comparing 63 cases that underwent PMX-DHP with 25 cases that did not.

## Results

The two groups did not differ significantly in age, APACHE II score, SOFA score, infection focus, and bacterial species. The 28-day mortality was 32% in the PMX-DHP group and 40% in the non-PMX-DHP group, but this difference was not significant. The ⊿CAI (the change in Cathecholamine Index 24 hours after PMX-DHP administration) and the infusion volume 24 hours after PMX-DHP administration were significantly different between the two groups. Multivariate analysis identified ⊿CAI and SOFA score as independent prognostic factors. Sub-analysis showed no significant difference in 28-day mortality in sepsis cases involving the lower-gastrointestinal tracts, but a trend for improvement in 28-day mortality was observed in sepsis cases where the infection focus could not be removed, such as pneumonia, and in severe sepsis cases whose APACHE II score exceeded 25.

## Conclusions

PMX-DHP may improve life prognosis of septic shock cases under certain conditions. We have to clarify the indication criteria for PMX-DHP and accumulate data by additional investigations of severe septic cases to pursue the possibilities for further improvements in life prognosis.

